# The Diagnostic and Prognostic Value of Reticulated Platelets in Ischemic Stroke: Is Immature Platelet Fraction a New Biomarker?

**DOI:** 10.3390/medicina61101887

**Published:** 2025-10-21

**Authors:** Fatih Cemal Tekin, Osman Lütfi Demirci, Emin Fatih Vişneci, Abdullah Enes Ataş, Hasan Hüseyin Kır, Hasan Basri Yıldırım, Çiğdem Damla Deniz, Demet Acar, Said Sami Erdem, Mehmet Gül

**Affiliations:** 1 Department of Emergency Medicine, Konya City Hospital, Konya 42020, Türkiye; osman_lutfi@hotmail.com (O.L.D.); drfatihvisneci@hotmail.com (E.F.V.); dr_demetacar@hotmail.com (D.A.); mehmetgul156@yahoo.com (M.G.); 2 Department of Radiology, Meram Faculty of Medicine, Necmettin Erbakan University, Konya 42090, Türkiye; aenesatas@gmail.com; 3 Department of Neurology, Meram Faculty of Medicine, Necmettin Erbakan University, Konya 42090, Türkiye; hasanhuseyinkir@gmail.com; 4 Department of Biochemistry, Konya City Hospital, Konya 42020, Türkiye; drhbyildirim@gmail.com (H.B.Y.); c.d.cetinkaya@gmail.com (Ç.D.D.); serdem1505@yahoo.com (S.S.E.)

**Keywords:** acute ischemic stroke, biomarkers, tests, diagnostic, emergency departments, emergency health services, prognosis

## Abstract

*Background and Objectives*: Ongoing efforts to develop early diagnostic tools for Acute Ischemic Stroke (AIS) point out the advantages of accessible biomarkers such as Immature Platelet Fraction (IPF). This is particularly important for emergency department (EDs), especially those that are overcrowded and have limited resources. The present study aimed to evaluate the diagnostic, prognostic, and therapeutic significance of IPF in patients with AIS presenting to the ED. *Materials and Methods*: This prospective case–control study was conducted in an ED. Participants aged 18-years and older who presented with complaints of numbness, weakness, diplopia or visual disturbances, speech or comprehension impairment, confusion, imbalance, impaired coordination and gait, or dizziness were included in the study. The diagnostic value of IPF in AIS and its relationship with short-term prognosis (STP) were investigated. Additional variables potentially associated with parameters such as infarct localization, number of lesions, affected hemisphere, main artery status, carotid status and treatment method were also analyzed. *Results*: The median age of the study participants was 67 years (Q1 = 54, Q3 = 76), with 48.9% (n = 88) being female and 51.1% (n = 92) male. Receiver operating characteristic curve analysis demonstrated that IPF was statistically significantly superior to other complete blood count parameters in the diagnostic evaluation of AIS. The diagnostic cutoff value of IPF for AIS was calculated as 2.45. An increase of 1 unit in IPF was found to raise the likelihood of AIS by 2.599 times. The Ratio of Red Cell Distribution Width (RDW) to IPF and NEU to IPF, mean corpuscular volume, and infarct volume were found to be significant predictors in STP assessment. *Conclusions*: Although not definitive alone, IPF may aid early stroke recognition, support treatment monitoring, and inform targeted therapies. The use of IPF, a biomarker that can be rapidly obtained, in the diagnosis of AIS is expected to yield beneficial outcomes in patient management, particularly in emergency departments and other clinical settings.

## 1. Introduction

Stroke-related morbidity and mortality have doubled over the past three decades, making stroke the second leading cause of death worldwide. With an aging global population, the burden of stroke is expected to increase further, with stroke-related deaths projected to rise by 50% by 2050. This alarming trend underscores the continuing need for efforts to mitigate the public health impact of stroke [1,2].

Acute Ischemic Stroke (AIS) is the most common type of stroke [3]. AIS has numerous modifiable and non-modifiable predisposing factors. Atherosclerosis and subsequent thromboembolic events are the primary contributors to its pathophysiology, as they result in impaired cerebral blood flow [4].

Platelets play a critical role in these thromboembolic processes. Numerous studies have focused on the role of platelets in AIS, their interactions with other physiological systems, and their implications for treatment strategies [5,6,7]. One emerging biomarker in this context is the Immature Platelet Fraction (IPF), which reflects the proportion of reticulated platelets (RP) in the bloodstream. RPs, which are rich in RNA, are immature platelets and have been associated with thromboembolic events. The IPF represents the ratio of these immature platelets to mature platelets, with a reported normal reference range of 1.1–6.1% of the total platelet count. IPF provides information regarding increased platelet production in the bone marrow. While it reflects a compensatory response to increased platelet destruction and demand, it also highlights the role of enzymatically active immature platelets in prothrombotic events. Several studies have proposed the potential utility of IPF in various thromboembolic conditions with different clinical presentations [8,9,10,11,12,13].

Prognostic biomarkers in AIS are of considerable importance, and recent studies in this field have gained attention. Blood parameters, due to their easy accessibility, straightforward interpretation, and rapid availability of results, may serve as valuable tools in the diagnosis, treatment, and prognostic follow-up of AIS. However, studies specifically addressing the relationship between AIS and IPF are relatively scarce in the literature. Although some investigations have explored the significance of reticulated platelets in AIS, clear and definitive evidence regarding their clinical utility is still lacking [12,14].

There is a strong expectation and hope that reticulated platelets and IPF may be utilized in the early warning, diagnosis, and monitoring of AIS [11,15]. However, studies conducted in this field remain quite limited. In particular, evaluations regarding its use as a diagnostic biomarker in the emergency department (ED) are almost nonexistent. The present study aimed to investigate the diagnostic and short-term prognostic value of IPF in AIS and to compare its performance with that of other biomarkers in patients presenting to EDs. The significance of this in the ED can be explained as follows: while physical examination findings are undoubtedly valuable, they may be subjective; therefore, in situations where access to or utilization of imaging modalities is limited, biomarkers may serve as an essential adjunctive tool for clinical decision-making. In this context, the present study is expected to provide important insights into new approaches and the potential role of biomarkers in the emergency department for patients with AIS.

## 2. Materials and Methods

This study was designed as a prospective case–control investigation. Based on sample size calculations (Student’s *t*-test, effect size = 0.5, α = 0.05, power = 90%), it was determined that 86 patients should be included in each group. Accordingly, the target sample consisted of 90 patients in both the AIS and control groups.

The age and gender characteristics of the cases were recorded. IPF and other laboratory parameters were analyzed in relation to the diagnosis of AIS. Factors influencing the radiological imaging findings of patients diagnosed with AIS (localization, number of lesions, affected hemisphere, main artery status, and carotid status), factors affecting treatment strategies (ward/ICU admission recommendation and treatment method), and determinants of short-term prognosis (STP) were evaluated. STP was determined by survival status (alive vs. deceased) within the first 30 days after diagnosis.

Voluntary participants aged 18 years and older who presented to the ED with complaints of numbness or weakness in the face, legs, or arms; diplopia or visual disturbances; speech or comprehension impairment; confusion; imbalance; coordination and gait disturbances; or dizziness were included in the study. Patients with a confirmed diagnosis of stroke (AIS) were assigned to the case group, while the control group consisted of patients without a stroke diagnosis. Patients diagnosed with transient ischemic attack (TIA) were excluded from the control group due to the challenges of follow-up and determining their short-term risk of stroke. Although the number of patients diagnosed with TIA was small, it was considered that the pathophysiology in TIA may resemble that of stroke, and therefore, IPF levels in this group could differ from those of both stroke and non-stroke controls, potentially acting as a confounding factor.

The diagnoses of patients included in the control group were benign paroxysmal vertigo (n = 16), chronic anemia (n = 6), seizure and postictal state (n = 13), hypoglycemia (n = 9), hyponatremia (n = 8), hypertensive encephalopathy in patients without prior hypertension (n = 2), dementia/Alzheimer’s disease (n = 10), psychiatric conditions such as hysteria, malingering, and psychosis (n = 6), vasovagal syncope (n = 7), facial paralysis (n = 10), and migraine attack (n = 3).

Exclusion criteria included pregnancy, absence of imaging data, concurrent or isolated hemorrhagic stroke, active bleeding, recent or ongoing radiotherapy and chemotherapy, radiotherapy-induced encephalitis or meningitis, presence of infection (fever, infectious focus, or elevated C-reactive protein), rheumatologic, autoimmune, or hematologic diseases, recurrent stroke history, and use of antiplatelet or anticoagulant therapy, as well as other embolic or thrombotic conditions (e.g., coronary, pulmonary, or deep vein thrombosis). Patients with a body mass index (BMI) ≥ 30 were also excluded. Additionally, it was confirmed that none of the participants had recently used non-steroidal or steroidal anti-inflammatory drugs, alcohol, or other agents that may affect platelet function (e.g., carbamazepine, valproic acid, or antibiotics such as ampicillin).

Blood sample and data collection were conducted between 15 February and 15 June 2025.

### 2.1. Sample Collection and Laboratory Analysis

Venous blood samples were collected upon ED admission using K2-EDTA vacutainer tubes (BD Diagnostics, Plymouth, UK). To ensure sample quality and reliability, all specimens were analyzed within 4 h of collection. Reticulocyte count, complete blood counts (CBC), and IPF measurements were performed using a Sysmex XN-1000 hematology analyzer (Sysmex, Kobe, Japan), with IPF determined via fluorescence flow cytometry according to the manufacturer’s protocol.

The CBC panel included the following biomarkers: white blood cells (WBC), red blood cells (RBC), hematocrit (HCT), mean corpuscular volume (MCV), mean corpuscular hemoglobin (MCH), neutrophils (NEU), leukocytes (LEU), platelet distribution width (PDW), red cell distribution width-standard deviation (RDW-SD), red cell distribution width-coefficient of variation (RDW-CV), immature platelet fraction (IPF), hemoglobin (HGB), platelet count (PLT), mean platelet volume (MPV), and plateletcrit (PCT).

### 2.2. Radiological Imaging Method

The definitive diagnosis of AIS was established using diffusion-weighted magnetic resonance imaging (DW-MRI) performed in the emergency department based on clinical suspicion. Infarct volume (IV) was calculated in cubic centimeters (cm^3^) from DW-MRI scans. The patients’ cerebral and cervical vascular lesions of the patients were evaluated using computed tomography (CT) angiography.

DW-MRI images of the patients were acquired on a 1.5T MAGNETOM Aera MRI system (Siemens, Erlangen, Germany). CT angiography images were obtained using a SOMATOM Perspective 128-slice CT scanner (Siemens, Erlangen, Germany).

### 2.3. Statistical Analysis

Statistical analyses were performed using IBM SPSS Statistics 21.0 (IBM Corp., Armonk, NY, USA) and jamovi (The jamovi project 2024, Version 2.6.26, Computer Software, Sydney, Australia). Categorical variables were presented as frequencies and percentages, while numerical data were expressed as mean ± standard deviation (SD) or as median and interquartile range (Q1–Q3). The chi-square (χ^2^) test was used for categorical comparisons. Normality of continuous variables was assessed using the Kolmogorov–Smirnov and Shapiro–Wilk tests.

For normally distributed variables, Student’s t-test was applied; for non-normally distributed data, Mann–Whitney U and Kruskal–Wallis H tests were used. Spearman’s correlation analysis was conducted for non-parametric relationships. Post hoc comparisons following Kruskal–Wallis and multi-group χ^2^ tests were adjusted using the Bonferroni correction.

## 3. Results

The median age of the study population was 67 years (Q1 = 54, Q3 = 76), with 48.9% (n = 88) female and 51.1% (n = 92) male participants. Comparative demographic and laboratory findings between the AIS and control groups are summarized in Table 1.

Table 2 presents the neuroimaging findings, treatment modalities, and STP outcomes of AIS patients. No statistically significant correlation was found between IPF levels and length of stay in the ward (*p* = 0.275) or intensive care unit (*p* = 0.614). Among patients admitted to the ward, 87.5% had no large vessel occlusion, and 55% had subcortical lesions. A significant association between IVs and treatment method was observed, with IVs being significantly higher in patients receiving interventional treatment compared to those undergoing non-specific management. Among patients without large vessel lesions, 77.3% (n = 58) were discharged, while 66.7% (n = 10) of those with large vessel lesions died. Cases who died had significantly higher IV values. Regarding lesion localization and STP, a statistically significant difference was observed, with MCA and subcortical lesions accounting for most outcomes. Specifically, 73.3% of deaths were associated with MCA infarctions, while 97.6% of patients with subcortical lesions were discharged. No statistically significant association was found between IPF and STP (mortality at 30 days) or the treatment method.

**Figure 1:**
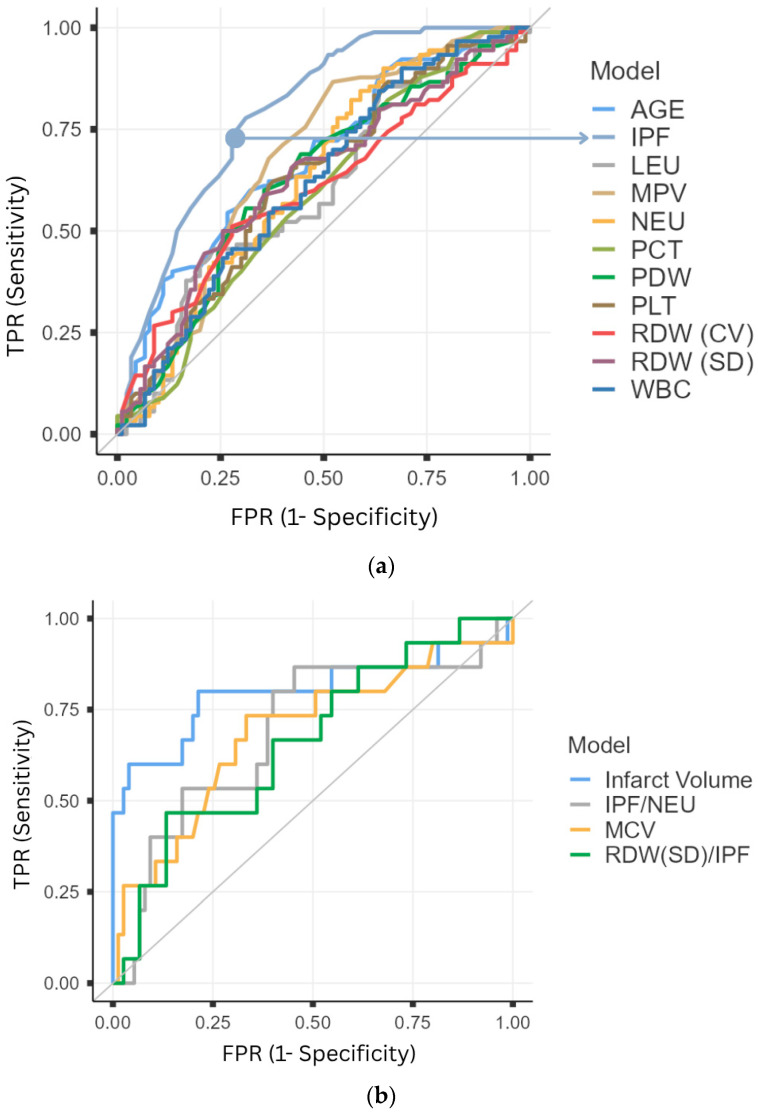
ROC Curve Analysis. (**a**) Diagnostic Discrimination; (**b**) Short Term Prognosis Prediction. TPR: True Positive Rate, FPR: False Positive Rate, IPF: Immature Platelet Fraction, MPV: Mean Platelet Volume, NEU: Neutrophil, PDW: Platelet Distribution Width, RDW (SD): Red Cell Distribution Width-Standart Deviation, RDW (CV): Red Cell Distribution Width-Coefficient of Variation, WBC: White Blood Cells, PCT: Plateletcrit, PLT: Platelet, LEU: Leukocytes, MCV: Mean Corpuscular Volum. Case group n = 90, control group n = 90.

ROC curve analysis was performed for the laboratory parameters that were found to be diagnostically significant (Figure 1), as shown in Table 1, and for those that demonstrated statistically significant differences with respect to STP, as presented in Table 2. IPF demonstrated significantly superior diagnostic performance compared to other CBC parameters (Delong’s Test: Age vs. IPF, *p* = 0.024; WBC vs. IPF, *p* < 0.001; NEU vs. IPF, *p* = 0.002; LEU vs. IPF, *p* < 0.001; PDW vs. IPF, *p* < 0.001; RDW-SD vs. IPF, *p* = 0.02; RDW-CV vs. IPF, *p* < 0.001; PLT vs. IPF, *p* < 0.001; MPV vs. IPF, *p* < 0.001; PCT vs. IPF, *p* < 0.001). The optimal cutoff value for IPF was 2.45, with an odds ratio (OR) of 0.12 (*p* < 0.001, 95% CI: 0.730–0.860), as shown in Table 3.

In terms of diagnosis, logistic regression analysis was performed using laboratory parameters that showed no multicollinearity based on correlation analysis: Age, IPF, NEU, RDW-SD, and LEU. The resulting model demonstrated that age (*p* = 0.008), IPF (*p* < 0.001), and NEU (*p* = 0.030) were statistically significant predictors. According to this model, a one-unit increase in age, IPF, and NEU was associated with a 1.038 (95% CI: 1.010–1.067), 2.599 (95% CI: 1.808–3.737), and 1.161 (95% CI: 1.015–1.327) fold increase in the likelihood of AIS, respectively (Table 4).

According to the logistic regression analysis performed with the variables found to be significant for STS, IV (*p* = 0.001) and MCV (*p* = 0.015) values constituted a significant model (Table 4). In this model, each 1-unit increase in IV increased the dead of patients diagnosed with AIS by 1.008 (95% CI: 1.003–1.013), whereas each 1-unit increase in MCV increased it by 1.159 (95% CI: 1.029–1.305) times.

## 4. Discussion

This article presents a single-center, prospective case–control study that investigates the value of RP and IPF as potential biomarkers for the diagnosis of AIS in patients with clinical suspicion of AIS, while also evaluating STP. The findings indicate that elevated IPF levels are diagnostically significant in AIS and superior to other hematological laboratory parameters in this regard. However, no significant relationship was observed between IPF alone and STP in AIS. Nonetheless, findings suggested that its combined use with inflammatory parameters such as the ratio of RDW to IPF and NEU to IPF might have potential significance.

Age is recognized as one of the most important non-modifiable risk factors for AIS. Recent reports indicate an increase in the number of stroke cases occurring in individuals under 70 years of age. This phenomenon may be associated with preventive measures implemented in older populations, improved health literacy leading to lifestyle modifications, and better management of chronic diseases. Nevertheless, the widely accepted view is that after the age of 55, the risk of AIS doubles with each decade, and nearly three-quarters of all AIS cases occur in patients over 65 years of age [1,14]. In the present study, the median age of AIS patients was determined to be 70 years (Q1 = 60, Q3 = 80), which is consistent with the literature and supports existing knowledge [15,16].

Gender is another important socio-demographic variable. Previous studies have demonstrated varying relationships between gender and stroke risk. Some reports suggest that while AIS incidence is higher in men under 55 years, the rates become similar between men and women after this age. More recent findings indicate that AIS incidence is higher in women under 30 years, predominates in men in middle age, and equalizes again in older age groups [17,18]. In this study, 56.7% of AIS patients were male. No statistically significant difference was observed between case and control groups in terms of gender distribution. To draw more definitive conclusions from these demographic variables, larger cohort and multicenter studies are required, considering the complex metabolic and hormonal interactions underlying age and gender.

The main focus of the current study was IPF, which can be regarded as a relatively novel parameter in clinical practice. Although some studies have addressed the role of reticulated platelets in AIS, there is still no clear and conclusive evidence regarding their clinical utility [19,20]. Given the role of RPs in thromboembolic events, particularly in cardiovascular conditions, there is strong anticipation that IPF may serve as a diagnostic and monitoring tool in AIS [11,12]. The present study supports this expectation. The results demonstrated that IPF outperformed other hematological parameters in diagnostic evaluation, as evidenced by the area under the ROC curve. Furthermore, DeLong’s test revealed that the diagnostic superiority of IPF over other laboratory parameters was statistically significant. The diagnostic cutoff value of IPF for AIS was determined to be 2.4. Accordingly, an IPF level above 2.4 may serve as a diagnostic warning for AIS, further emphasizing the potential role of RPs in AIS pathogenesis.

Beyond IPF, other laboratory parameters found to be diagnostically significant in AIS included age, MPV, WBC, NEU, LEU, PDW, RDW-SD, RDW-CV, PLT, and PCT. Although numerous confounders and risk factors were excluded, considering the coexistence of multiple comorbidities in ED patients, it would not be appropriate to claim that these parameters alone are sufficient for AIS diagnosis. Nevertheless, logistic regression analysis in this study demonstrated that age and NEU, when combined with IPF, may form a useful diagnostic model. The model indicated that AIS risk increased significantly in patients over 62 years of age, a value close to those used in some stroke scoring systems [21]. Another significant parameter in the model was NEU (cutoff ≥ 6.94), which supports numerous studies describing the strong association between inflammatory processes and AIS, as well as changes in neutrophil activity shortly after stroke onset [22,23,24]. Therefore, this diagnostic model provided more efficient results than IPF alone. This finding highlights the importance of using clinical decision-making scoring systems in the ED, rather than relying on a single parameter [25]. Its implication for the emergency department is even greater. Diagnostic adjuncts may help prevent diagnostic uncertainty and time loss during patient transfers in settings where time is critical and resources are limited, ultimately contributing to faster and more effective patient management. Incorporating IPF into such scoring systems or machine learning models may be of considerable value. Moreover, supportive laboratory markers are particularly important in settings lacking MRI facilities, where rapid AIS diagnosis is critical. Given the high burden of AIS in terms of mortality, morbidity, and over 160 million disability-adjusted life-years (DALYs) lost worldwide [1], even a modest improvement in early diagnosis could have major clinical implications.

As a biomarker, IPF alone did not yield statistically significant results in predicting short-term prognosis (STP), length of hospital stay, the need for ward or intensive care unit admission, or treatment strategies (thrombolysis, thrombectomy, or non-specific treatment) in patients with AIS. However, given the evidence in the literature linking inflammatory processes to prognosis [23,24,26,27], this study found that while NEU and RDW alone were not associated with STP, the combined ratios NEU/IPF and RDW (SD)/IPF were significant predictors of short-term prognosis. Considering the role of neutrophils and platelets in activation, adhesion, aggregation, and infarct site activity during the inflammatory process [5], future studies should focus on the interactions between RPs and immune cells and the prognostic value of IPF in combination with other inflammatory parameters. Although this study did not demonstrate a significant association, previous research has suggested that IPF may play a role in assessing treatment response, particularly with antiplatelet agents, and in therapeutic monitoring [28,29]. Co and Yu also reported that early neurological deterioration in AIS patients was associated with IPF levels exceeding 5%, which emphasizes the possible role of IPF in prognosis, though the current study suggests it may be premature to make such conclusions. Further research is needed to evaluate the role of IPF in clinical follow-up, prognosis, and treatment monitoring in AIS.

When other parameters beyond IPF were examined in relation to prognosis and treatment strategies, infarct volume (cm^3^) was found, in line with previous literature [30,31], to influence ward/ICU admission, treatment choice, and short-term prognosis. This association was mostly observed in middle cerebral artery (MCA) infarcts. Additionally, MCV was significantly associated with ward/ICU admission and short-term prognosis, consistent with earlier studies [32]. ROC curve analysis of prognostic factors revealed that an infarct volume greater than 34.11 cm^3^ and MCV levels above 90.5 were significantly associated with poor prognosis.

## 5. Limitations and Future Directions

This study primarily addresses the results obtained from a single-center experience. Although the sample size in this study was relatively limited, similar studies with comparable sample sizes are available in the literature. Nevertheless, larger-scale studies are needed to fully elucidate risk factors and potential confounders. Comparative analyses regarding AIS risk factors and confounders were not performed in this study. At the same time, since many patients present to the emergency department with various preliminary diagnoses, and the study aimed to highlight the importance of rapid biomarkers such as IPF that could strengthen the diagnosis of AIS among these cases, the control group included patients with different non-AIS conditions. Although this led to a limited number of cases in each diagnostic subgroup, preventing detailed comparative analyses, this design was not inappropriate, as it aligned with the study’s aim of exploring the potential use of IPF in the emergency department setting. To minimize potential misinterpretation of IPF results, broad exclusion criteria were applied to reduce this limitation as much as possible. In the Methods section, these confounding factors are detailed comprehensively. The identification of confounding factors was based on a literature review to determine variables that could increase platelet demand due to consumption, or suppress or enhance platelet production. Considering the multitude of risk factors, such analyses may only be feasible through multicenter studies with extended data collection periods. Because determining different projections of IPF according to risk factors requires grouping and analyzing a large number of patients diagnosed with conditions such as diabetes, hypertension, and obesity. For a parameter that is not yet clearly established for clinical use, this issue supported by the evidence from the present study may be considered a potential topic for future research. Rather than relying on a single parameter, incorporating IPF into scoring systems or combined models, as proposed in our study, may represent a potential solution to this challenge. Considering that this study was conducted in the Emergency Department and that the parameter in question would be applied to patients with multiple confounding and risk factors, it was deemed more appropriate rather than using a parameter that may vary according to different risk factors to define exclusion criteria, as was done in this study. Patients diagnosed with transient TIA were excluded from the study due to the necessity of clinical follow-up. At this point, certain questions may arise in the minds of the readers. Including TIA cases in the control group would have been inappropriate given the group’s dynamics. Moreover, as the number of TIA cases was insufficient, comparison with AIS patients was avoided to prevent misleading conclusions regarding the role of RPs, since these two groups share similar pathophysiology. Future research is warranted to directly compare IPF between TIA and AIS. Such studies may also shed light on the potential of RP-focused therapeutic strategies in preventing AIS. In addition, hemorrhagic stroke cases were excluded, as hemorrhagic events are believed to increase RP levels due to heightened consumption and compensatory demand. Furthermore, hemorrhagic stroke can be more readily diagnosed using CT scans, which are more accessible than MRI; thus, the focus of this study was placed on the role of RP in AIS. Taken together, this study provides preliminary data on the role of RP in AIS. In the future, studies evaluating IPF in both AIS and TIA populations are required, as they may provide further evidence clarifying the diagnostic and prognostic value of this biomarker.

## 6. Conclusions

In this study, a diagnostic cutoff value for IPF (2.4%) was identified, and the diagnostic performance of IPF was found to improve when evaluated in combination with age and NEU counts. These findings suggest that IPF may serve as a supportive tool for the diagnosis of AIS in the emergency department. Based on the short-term prognostic outcomes of the study, it can be interpreted that increases in MCV and infarct volume are associated with reduced survival. However, evidence was obtained suggesting that IPF alone may serve as an indicator of survival. Nonetheless, the association of inflammatory parameters (RDW, NEU) in relation to IPF with prognosis suggests that it may be premature to draw definitive conclusions regarding the role of RPs in AIS prognosis.

Biomarkers are potential candidates to play a significant role in the diagnosis and monitoring of AIS. Hematological parameters are particularly valuable due to their accessibility, ease of interpretation, and rapid turnaround times. In the context of overcrowded emergency departments and the need for rapid clinical decision-making, the utility of such biomarkers becomes even more significant. In addition to supporting previous evidence on IPF, this study focused on its potential utility in the ED. It is anticipated that the findings will inform and support future investigations on the use of biomarkers in the diagnosis and follow-up of AIS.

## Figures and Tables

**Table 1:** Variables Related to the Case and Control Groups and Results of Statistical Comparisons Between the Groups.

**Variables**		**Unit**	**Case**		**Control**		
			**n**	**%**	**n**	**%**	***p*-Value**
Gender	Female		51	56.7	41	45.6	*p* = 0.136 χ^2^ = 2.223
	Male		39	43.3	49	54.4	
			**Median**	**Q1–Q3**	**Median**	**Q1–Q3**	
Age		Year	70	60–80	60	48–72	*p* < 0.001 U = 2529.5
Laboratory Parameters	WBC	10^9^/L	9.05	7.51–12.49	8.30	6.81–10.10	*p* = 0.006 U = 3090.5
	RBC	10^12^/L	4.83	4.47–5.26	4.78	4.37–5.15	*p* = 0.471 U = 3798.0
	HCT	%	43.00	38.78–46.13	41.85	38.40–44.68	*p* = 0.164 U = 3563.5
	MCV	fL	88.10	85.38–93.30	87.80	83.73–91.33	*p* = 0.298 U = 3686.0
	MCH	pg	28.80	27.78–30.05	28.80	27.23–30.20	*p* = 0.792 U = 3958.0
	NEU	10^9^/L	6.12	4.72–9.75	5.27	4.02–6.51	*p* = 0.020 U = 2979.5
	LEU	10^9^/L	1.78	1.11–2.38	1.98	1.44–2.86	*p* = 0.019 U = 0.3232
	PDW	fL	12.25	10.93–13.33	11.15	10.00–12.43	*p* = 0.040 U = 3036.0
	RDW (SD)	%	44.40	41.95–48.70	42.25	40.55–46.28	*p* = 0.002 U = 2990.0
	RDW (CV)	%	14.00	13.20–15.13	13.30	12.60–14.60	*p* = 0.016 U = 3210.0
	IPF	%	3.05	2.08–5.00	1.65	1.20–2.40	*p* < 0.001 U = 1663.0
			**Mean ± SD**		**Mean ± SD**		
Laboratory Parameters	HGB	g/dL	13.72 ± 2.09		13.46 ± 1.98		*p* = 0.378 t = 0.885
	PLT	10^9^/L	237.11 ± 84.09		270.04 ± 73.19		*p* = 0.007 t = −2.705
	MPV	fL	10.56 ± 1.00		9.94 ± 0.82		*p* < 0.001 t = 4.556
	PCT	%	0.25 ± 0.78		0.28 ± 0.66		*p* = 0.025 t = −2.261

WBC: White Blood Cells, RBC: Red Blood Cells, HCT: Hematocrit, MCV: Mean Corpuscular Volum, MCH: Mean Corpuscular Hemoglobin, NEU: Neutrophil, LEU: Leukocytes, PDW: Platelet Distribution Width, RDW (SD): Red Cell Distribution Width-Standart Deviation, RDW (CV): Red Cell Distribution Width-Coefficient of Variation, IPF: Immature Platelet Fraction, HGB: Hemoglobin, PLT: Platelet, MPV: Mean Platelet Volume, PCT: Plateletcrit, Chi-Square: χ^2^, U: Man Whitney U, t: Student-*t* Test. Statistically significant values are denoted in bold. Case group n = 90. Control group n = 90.

**Table 2:** Imaging Study Characteristics, Treatment Strategies, and Prognosis in Patients.

Variable		n	%	IPF	Statistically Associated Parameters
Localization	Lacunar and Subcortical	41	45.6	*p* = 0.923 H = 161.0	Service/ICU Recommendation (*p* = 0.006 χ^2^ = 10.28), Short Term Prognosis (*p* < 0.001 χ^2^ = 27.46)
	Watershed	21	23.3		
	ACA	7	7.8		
	MCA	11	12.2		
	Basilar and PCA	6	6.7		
Number of Lesions	Single	65	72.2	*p* = 0.675 U = 766.0	Reticulocytes (*p* < 0.001, U = 71.5) *
	Multiple	25	27.8		
Affected Hemisphere	Right	39	43.3	*p* = 0.063 H = 5.541	Absent
	Left	39	43.3		
	Bilateral	12	13.4		
Main Artery Status	Occlusion Absent	63	70.0	*p* = 0.526 U = 775.5	Service/ICU Recommendation (*p* = 0.001 χ^2^ = 10.50), Short Term Prognosis (*p* < 0.001 χ^2^ = 11.52 )
	Occlusion Present	27	30.0		
Carotid Status	Normal	23	25.6	*p* = 0.854 H = 0.782	Absent
	Plaque without stenosis	40	44.4		
	Significant stenosis	17	18.9		
	Occlusion	10	11.1		
Service/ICU Recommendation	Service	40	44.4	*p* = 0.798 U = 968.5	MCV (*p* = 0.045 U = 753.0), Localization (*p* = 0.006 χ^2^ = 10.28), Main Artery Occlusion (*p* = 0.001 χ^2^ = 10.50), Infarct Volume (*p* < 0.001 U = 541.0)
	ICU	50	55.6		
Treatment Method	Thrombolytic	11	12.2	*p* = 0.211 H = 3.110	Infarct Volume (*p* = 0.004 H = 10.95)
	Interventional	20	22.2		
	Non-Specific	59	65.6		
Short Term Prognosis (Initial 30 days)	Alive	75	83.3	*p* = 0.132 U = 423.5	MCV (*p* = 0.023 U = 352.0), NEU/IPF (*p* = 0.021, U = 350.0), RDW (SD)/IPF (*p* = 0.048 U = 380.0), Reticulocytes (*p* = 0.015 U = 39.0) *, Main Artery Occlusion (*p* < 0.001 χ^2^ = 27.46), Localization, Infarct Volume (*p* < 0.001 U = 225.0)
	Deceased	15	16.7		

IPF: Immature Platelet Fraction, NEU: Neutrophil, RDW (SD): Red Cell Distribution Width-Standart Deviation, ICU: Intensive Care Unit, MCV: Mean Corpuscular Volume, ACA: Anterior cerebral artery, MCA: Middle cerebral artery, PCA: Posterior cerebral artery, U: Man Whitney U Test, t: Student-*t* Test, Chi-Square: χ^2^, H: Kruskal–Wallis H Test. Statistically significant values are denoted in bold. * Case group n = 90, control group n = 90. There was missing data in the parameter Reticulocyte Count. Therefore, the effect of reticulocyte was evaluated on 41 patients.

**Table 3:** Variables Related to the and Control Groups and Results of Statistical Comparisons Between the Groups.

Variables	Area	SE	*p*	95% CI		Cutoff	Sensivity	Specifity	PPV	NPV
				Lower	Upper		(%)	(%)	(%)	(%)
Diagnostic Significance in Acute Ischemic Stroke										
IPF (%)	0.795	0.033	<0.001	0.730	0.860	≥2.45	68.89	77.78	75.61	71.43
AGE (Year)	0.679	0.040	<0.001	0.601	0.757	≥62	73.33	54.44	61.68	67.12
MPV (fL)	0.681	0.040	<0.001	0.602	0.760	≥10.65	47.78	86.67	78.18	62.40
NEU (10^9^/L)	0.632	0.042	0.002	0.550	0.714	≥6.94	43.33	82.22	70.91	59.20
PDW (%)	0.625	0.042	0.004	0.543	0.707	≥11.35	68.89	55.56	60.78	64.10
RDW-SD (%)	0.631	0.042	0.002	0.549	0.712	≥42.15	74.44	50.00	59.82	66.18
WBC (10^9^/L)	0.618	0.042	0.006	0.537	0.700	≥11.83	31.11	90.00	75.68	56.64
RDW-CV (%)	0.604	0.042	0.016	0.521	0.687	≥13.35	72.22	51.11	59.63	64.79
PLT (10^9^/L)	0.374	0.042	0.003	0.292	0.455	≤254	63.33	61.11	61.96	62.50
PCT (&)	0.409	0.042	0.036	0.326	0.493	≤0.22	34.44	82.22	65.96	55.64
LEU (10^9^/L)	0.399	0.042	0.019	0.316	0.482	≤2.53	80.00	42.22	58.06	67.86
Short-Term Prognostic Significance in Acute Ischemic Stroke										
Infarct Volume (cm^3^)	0.800	0.082	<0.001	0.640	0.960	≥34.11	80.00	78.70	42.80	95.70
MCV (fL)	0.687	0.082	0.023	0.527	0.847	≥90.5	73.30	66.70	30.60	92.60
IPF/NEU	0.311	0.080	0.021	0.155	0.467	≤0.51	86.65	54.67	27.66	95.35
RDW(SD)/IPF	0.662	0.076	0.048	0.514	0.810	≥1.95	86.68	54.67	27.66	95.35

RDW-SD: Red Cell Distribution Width-Standart Deviation, RDW-CV: Red Cell Distribution Width-Coefficient of Variation, WBC: White Blood Cells, PLT: Platelet, PCT: Plateletcrit, LEU: Leukocytes, MCV: Mean Corpuscular Volume, SE: Standart Error, CI: Confidence Interval, PPV: Positive Predictive Value, NPV: Negative Predictive Value. Statistically significant values are denoted in bold. Case group n = 90, control group n = 90.

**Table 4:** Results of Logistic Regression Analysis for Variables Showing Statistically Significant Differences in Terms of Diagnostic and Prognostic Evaluation.

Variables	B	SE	Wald	df	*p*	Exp(B)	95% CI for EXP(B)	
							Lower	Upper
Diagnostic Model ^1^								
Age (Year)	0.037	0.014	7.069	1	0.008	1.038	1.010	1.067
IPF (%)	0.955	0.185	26.587	1	<0.001	2.599	1.808	3.737
NEU (10^9^/L)	0.015	0.068	4.735	1	0.030	1.161	1.015	1.327
RDW-SD (%)	0.070	0.038	3.333	1	0.068	1.073	0.995	1.157
LEU (10^9^/L)	−0.045	0.175	0.067	1	0.796	0.956	0.678	1.347
Short-Term Prognostic Model ^2^								
Infarct Volume (cm^3^)	0.008	0.002	11.362	1	0.001	1.008	1.003	1.013
MCV (fL)	0.148	0.061	5.945	1	0.015	1.159	1.029	1.305
IPF (%)	0.026	0.327	0.006	1	0.936	1.027	0.541	1.947
IPF/NEU	−0.737	1.184	0.387	1	0.534	0.479	0.047	4.875
IPF/LEU	−0.415	0.260	2.542	1	0.111	0.660	0.397	1.100

IPF: Immature Platelet Fraction, NEU: Neutrophil, RDW (SD): Red Cell Distribution Width–Standard Deviation, LEU: Leukocytes, MCV: Mean Corpuscular Volume, SE: Standard Error, CI: Confidence Interval, PPV: Positive Predictive Value, NPV: Negative Predictive Value. Statistically significant values are denoted in bold. Case group n = 90, control group n = 90. ^1^ Model performance indicators: Control = 0, Case = 1; Omnibus Tests of Model Coefficients *p* < 0.001; Nagelkerke R^2^ = 0.485; Hosmer–Lemeshow Test *p* = 0.963; Overall Correct Classification = 78.9%; Sensitivity = 74.4%; Specificity = 83.3%; PPV = 81.7%; NPV = 76.5%; Accuracy = 78.9%. ^2^ Model performance indicators: Discharged = 0, Deceased = 1; Omnibus Tests of Model Coefficients *p* < 0.001; Nagelkerke R^2^ = 0.571; Hosmer–Lemeshow Test *p* = 0.659; Overall Correct Classification = 78.9%; Sensitivity = 92.1%; Specificity = 81.8%; PPV = 97.3%; NPV = 60.0%; Accuracy = 91.1%.

## Data Availability

Data is unavailable due to privacy and ethical restrictions. Ethical approval and informed consent for sharing the study data with third parties were not obtained. However, the data are stored in both private dataset and institutional database. To request access to the data, please contact the Konya City Hospital officials at konyash.etik@saglik.gov.tr or reach out to the corresponding author.

## Abbreviations

The following abbreviations are used in this manuscript:

AIS	Acute Ischemic Stroke
ED	Emergency Department
IPF	Immature Platelet Fraction
IV	Infarct volume
MCV	Mean Corpuscular Volume
NEU	Neutrophils
RDW	Red Cell Distribution Width
ROC	Receiver Operating Characteristic
RP	Reticulated Platelets
